# The development of robotics courses for young children under vector space model

**DOI:** 10.1371/journal.pone.0293397

**Published:** 2023-10-30

**Authors:** Yuanyuan Zuo, Lixuan Che, Liping Zhang

**Affiliations:** 1 School of Preschool Education, Jinan Vocational College, Jinan, China; 2 School of Cultural Innovation, Weifang Vocational College, Weifang, China; 3 Weifang Vocational College, Weifang, China; Sunway University, MALAYSIA

## Abstract

Robotics education is important in training children’s thinking, practical, and innovation abilities. It is significant to stimulate children’s interest in learning and improve their learning quality. The existing research has not paid attention to the application of robotics education in children. It is necessary to stimulate children’s interest in learning. This paper will take senior kindergarten students as the research object. It analyzes the application of the Vector Space Model (VSM) in robotics course development. The research and development of children’s robotics courses incorporating Artificial Intelligence technology are based on the survey results of robotics courses offered by 38 kindergartens in Baoji City. An automatic document classification system based on VSM is designed to assist in compiling robotics teaching textbooks. Finally, the system performance is tested. The results show that about 24% of kindergartens offer robotics courses, and 76% do not. Besides, 70.14% of teachers support the establishment of children’s robotics courses. The classification effect of the VSM system is better than that of Chinese documents. This system performs better than the automatic document classification system based on Term Frequency–Inverse Document Frequency. Its classification accuracy, recall, and F1 value are all above 85%. The development of the robotics course provides a better teaching environment for teaching young children about AI and robots. The robotics education discussed in this paper is a hot spot in the current curriculum reform and is of great significance to the development and innovation in early childhood education.

## 1. Introduction

Since the 21st century, Artificial Intelligence (AI) has entered a period of rapid development, creating technical conditions for developing intelligent robots [1], and robotics education has gradually received significant attention. Robotics education can exercise students’ thinking, manipulative, and innovation abilities [2]. It plays a positive role in promoting the growth of students. Modern robotics education mainly aims at primary and secondary school students [3]. However, senior kindergarten students can also learn robotics courses. Their learning focuses on the construction of robots, which lays the foundation for further learning in subsequent robotics courses. Service robots have many applications, including maintenance, repair, transportation, cleaning, security, rescue, monitoring, and other work. After years of collection and sorting, the International Federation of Robotics has given a preliminary definition of service robots. A service robot is a semi-autonomous or fully autonomous robot. It can complete the service work beneficial to human health but does not include the production equipment. The goal of robotics education can only be achieved if children can learn useful knowledge in robotics courses. Robotics education can cultivate children’s abilities in many aspects and solve problems.

Robotics education is an effective tool to cultivate children’s creativity. It can stimulate children’s curiosity about the things around them and have a profound impact on children’s creativity, hands-on ability, and communication and collaboration. However, there are many problems in robotics education in China, such as unclear curriculum standards and lack of relevant guidance. Based on this, this paper believes that preschool education, as an enlightenment education, should also let children understand robots, make good use of robots, and innovate robots to lay a good foundation for cultivating innovative talents in the future. This paper aims to improve the curriculum design of robotics education and carry out robotics education to tap the potential of early childhood development.

According to the above results, 38 kindergartens are randomly selected in Baoji City to investigate the current situation of robotics courses for children. Senior kindergarten students are taken as the research object. In all aspects of developing robotics courses for young children, AI technology is integrated into the development process, and robotics courses for advanced kindergartens are developed. The application of Vector Space Model (VSM) in robotics curriculum development is analyzed. In addition, an automatic textbook classification system based on spatial vector patterns has been established. To a certain extent, this paper provides research experience for developing robotics courses for children and has specific practical significance. Meanwhile, developing a robotics course can provide a good teaching environment for children to understand and learn AI and robot service automation. The robotics education curriculum developed here can better stimulate children’s interest and learning ability. Young children maintain a high degree of concentration and persistence in the things they are interested in. In this way, it promotes the development of students’ comprehensive ability and cultivates innovative talents.

## 2. Literature review

VSM is a commonly used technique in natural language processing, which can be used in text classification, information retrieval, and other fields. In the development of robotics courses for young children, the model can be applied to the process of understanding and generating natural language. Specifically, the task of language processing in young children can be thought of as the process of converting natural language into vectors. In this process, each word in a young child’s conversation can be represented as a vector. For a child robot course, all conversations and questions can be expressed as vectors, and these vectors can be used to build a VSM. In this model, it is possible to determine which problems are more relevant to which conversations by calculating the cosine similarity between vectors.

In recent years, significant achievements have been made in robotics education. Huijnen et al. (2017) summarized the robots currently used in the Autism Spectrum Disorder treatment and education of children and pointed out their goals [4]. Jung and Won (2018) systematically and thematically reviewed the existing literature on robotics education using robotics kits (non-social robots) for children (preschool and kindergarten to grade five). In addition, they pointed out that in previous studies, robotics education was only used to support other disciplines or limitations of a tool for Science, Technology, Engineering, and Mathematics (STEM) education [5]. Barnes et al. (2020) combined STEM with art and design. An extracurricular project- "Children-Robot Theatre" was built for children in rural primary schools. It provided research experience for STEAM education using robots and drama in science, art, and education [6]. Yang et al. (2020) used VOSviewer software to visually analyze the research literature on robotics education of the Web of Science in the past ten years. Additionally, they proposed that the research hotspots of robotics education mainly focused on cultivating students’ computational thinking, teaching practice education, and teaching tools of robots and the environment [7]. Kalogiannakis et al. (2021) [8] studied the impact of students’ acceptance of robotics courses on the development of skills, such as computational thinking. They analyzed the correlation between computational thinking and increased programming motivation. Research showed that more use of BBC micro:bit devices and their teaching strategies was needed to improve student learning. Tzagkaraki et al. (2021) [9] noted in their study that educational robots were an innovative and useful tool. It positively influenced critical thinking, computational thinking, problem solving, algorithmic thinking, creativity, and collaboration.

By summarizing the existing research, it can be found that robotics education is different from the traditional teaching form. The teaching form that emphasizes multiple experience has reference significance for other scientific teaching practices and promotes the improvement of teaching mode and strategy. However, in the development of children’s robot courses, there is no more systematic design plan. Here, an automatic textbook classification system based on spatial vector mode will be established to realize the overall development of robotics courses.

## 3. Theory and research methods

### 3.1 The integration of AI and the teaching of robotics courses for young children

**Fig 1:**
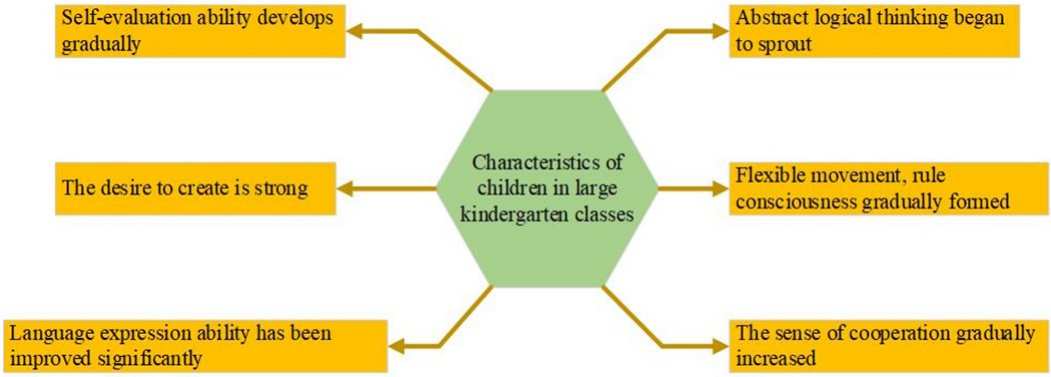
Ability learning that senior kindergarten students should accept.

The robotics courses are mainly for children to understand the principles of robots and learn how to program the robots to complete corresponding actions. It can not only improve children’s ability of hands-on and innovation but also cultivate children’s fun in science and enhance their ability to discover, think, and solve problems. The robotics courses can enable children to actively work with others. The curriculum content of children’s robots is generally designed with activities or games, which can enable children to learn in "playing" and make them interested in learning. The research object is senior kindergarten students, and its characteristics are shown in Fig 1.

The core of the development of AI and robotics courses is intelligent robot-related technologies. Through the development of these robotics courses, an innovative educational platform can be provided for young children. Although AI and robot are closely related, their focuses differ. Schools can organically integrate the two related technologies, integrate relevant knowledge, realize the combination of theory and reality, and accomplish the educational goals of robotics courses for young children by developing AI and robotics courses. Intelligent robots are developed with the introduction of "AI." Its fundamental purpose is to allow computers to simulate human thinking. An intelligent robot is a machine system that completely simulates human beings in terms of perception, thinking, and effects. The technical requirements of intelligent robots have the following three points.

The first is the recognition process, which converts the information input from the outside world into conceptual, logical information. It converts dynamic and static images, sounds, voice, text, touch, taste, and other information into formalized conceptual, logical information. The second is the process of intelligent computing. Input information stimulates self-learning, information retrieval, logical judgment, and decision-making and produces corresponding responses. The third is the control process, which converts the output response into body movements and media information.

According to the above description of the technology of intelligent robots, when developing young children’s robotics courses, robots have intelligent sensory, movement, and thinking functions to meet the intelligent requirements of robots for children’s robotics course development and the needs of the course. Young children’s intelligent robots must have the following three elements.

First, the sensory element is used to understand the state of the surrounding environment. Sensing elements include non-contact sensors that can sense vision, proximity, and distance and contact sensors that can sense power, pressure, and touch. These elements are equivalent to the facial features of the human eyes, nose, and ears. Besides, their function can be achieved using electromechanical elements, such as cameras, image sensors, ultrasonic transducers, lasers, conductive rubber, piezoelectric elements, pneumatic components, and travel switches.

Second, the moving element makes reactive actions to the outside world. For the moving element, the intelligent robot needs a trackless moving mechanism to adapt to different geographical environments, such as flat ground, steps, walls, stairs, and ramps. Their functions can be achieved by moving mechanisms, such as wheels, rails, feet, suction cups, and air cushions. During the movement, the movement mechanism should be controlled in real-time, including not only the position control but also the intensity control, the position and intensity mixed control, and the expansion rate control.

Third, the thinking element is necessary. Based on the information obtained by the sensory element, think about what action to take. The thinking element of an intelligent robot is the key of the three elements, and it is the essential element humans endow with the robot. The elements of thinking cover intellectual activities, such as judgment, logical analysis, and understanding. These intellectual activities are essentially an information processing process that computers can do.

### 3.2 VSM

In the compilation of children’s robotics textbooks, an automatic document classification system based on VSM is designed to promote children-oriented robotics courses. VSM was proposed by Salton and others in the early 1970s [10]. It was first applied to the SMART text retrieval system. VSM refers to the simplified processing of text content and conversion to vector operations in vector space. The similarity between semantics is expressed by the similarity of vectors in space [11]. A document is usually composed of words or phrases [12]. In the VSM, these can be represented by the feature [13].

The feature is the smallest unit in the VSM [14]. A document can be considered as the collection of all the features. It is shown in Eq (1).

$$Document=D\left(t_{1},t_{2},\cdots ,t_{k},\cdots ,t_{n}\right)$$ (1)

*n* is the number of the feature. *t*_*k*_ is the feature, and 1 ≤ *k* ≤ *n*.

In the document, each feature will be given a certain weight to indicate its importance, and its weight is shown in Eq (2).

$$D_{w}=D\left(w_{1},w_{2},\cdots ,w_{k},\cdots ,w_{n}\right)$$ (2)

*w* represents the weight, *w*_*k*_ shows the weight of the feature *t*_*k*_, and 1 ≤ *k* ≤ *n*.

There is no duplication between the feature in document *D*. Regardless of the document’s internal structure, there is no sequential relationship in the arrangement of the feature, and the feature is regarded as an n-dimensional coordinate system with a weight of coordinate value. The VSM of document *D* can be represented by Eq (3).

$$D=D\left(t_{1},w_{1};t_{2},w_{2};\cdots ;t_{k},w_{k};\cdots t_{n},w_{n}\right)$$ (3)

*w*_*k*_ can represent the frequency that *t*_*k*_ appears in document *D*, and its functional relationship can be expressed as:

$$w_{k}\left(d\right)=\varphi \left(tf_{k}\left(D\right)\right)$$ (4)

The common functions φ are expressed in Eqs (5)–(7).

Boolean function:

$$\varphi =\left\{\begin{array}{c} 0,tf_{k}\left(D\right)=0,\\ 1,tf_{k}\left(D\right)=1 \end{array}\right.$$ (5)

Square Root function:

$$\varphi =\sqrt{tf_{k}\left(D\right)}$$ (6)

Logarithmic function:

$$\varphi =\text{log}\left(tf_{k}\left(D\right)+1\right)$$ (7)

### 3.3 Investigation of the current situation of children’s robotics courses

The investigation and study of the current situation of the children’s robotics courses can help understand the existing problems in the current offer of the children’s robotics courses so that the research can be more in line with the actual situation. Also, it provides reference materials for the research. This paper takes Baoji City as an example. A random selection of 38 kindergartens is used to investigate the current situation of robot courses for young children. The research content is in line with the ethical standards established by the ethics committee.

The questionnaire is mainly for teachers. The content includes four dimensions: the kindergarten’s teaching objectives, curriculum arrangement, whether to offer robotics courses for children, and the teacher’s attitude towards children’s robotics courses.

The teaching objectives of kindergartens include living habits, self-care ability, manipulative ability, communication ability, presentation skill, thinking ability, innovation ability, problem-solving ability, and teamwork ability. Teachers who participate in the questionnaire can select five of these projects and sort them according to their importance. Items 1 to 5 are scored by 5, 4, 3, 2, and 1, respectively. Then, the item’s score is calculated by Eq (8).

$$S=\frac{\sum_{i=1}^{n}\;score}{n}$$ (8)

*S* means the comprehensive score, *n* is the number of statistical questionnaires, *i* is the i questionnaire, and *score* shows the score of the i questionnaire.

When the reliability test is performed with SPSS26.0, the internal consistency α coefficient is higher than 0.70, and the α coefficient at all levels is higher than 0.70. SPSS26.0 software is used to check the reliability of the measurement table, and the reliability of the total table and the reliability index of each dimension show that its reliability is high. As for the curriculum arrangement, it is mainly to investigate the courses currently offered by the kindergarten, which is filled in by the teacher according to the actual situation and counted. Fill in "Yes" or "No" according to whether it offers robotics courses in kindergartens. The attitude towards children’s robotics courses is divided into five options: "very necessary," "more necessary," "general," "relatively unnecessary," and "very unnecessary." In this survey, 312 questionnaires were issued, and 291 were recovered, with a recovery rate of 93.26% and 278 valid questionnaires with an efficiency of 89.10%.

### 3.4 The development process of the intelligent robotics course

Curriculum development [15] refers to determining the course’s learning goals according to the learners’ learning needs. According to the curriculum goals, the curriculum content of a particular subject, the curriculum implementation method, and the curriculum evaluation content are revised, and the curriculum goal is realized.

Firstly, the curriculum goal is determined. The main goal of kindergarten education is to cultivate children’s innovative spirit, improve children’s ability to find, analyze and solve problems, and cultivate their awareness of teamwork. Robotics programming courses are relatively complex, and there is a specific difficulty for children to understand, so the task of the children’s robotics courses is to cultivate robotics construction ability. So, children are interested in the structure and balance of machinery and other related knowledge. Children can learn to apply classroom knowledge to real life and explore knowledge in real life.

**Fig 2:**
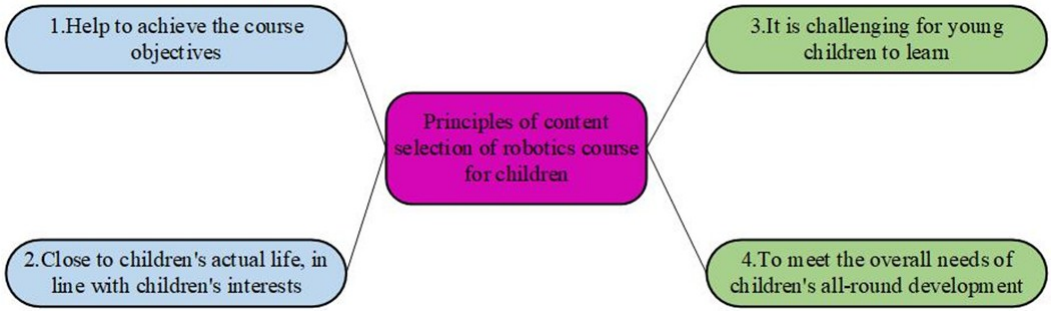
Principles of content selection of robotics courses for children.

Secondly, the curriculum content is selected and designed. Curriculum content is an important means for teachers to achieve teaching goals, and the setting of curriculum content should be consistent with the planning of curriculum goals. Interest is the best teacher. Especially for children, their self-control is worse than that of teenagers, youth, and adults. If the content taught by teachers cannot stimulate their interest in learning, the teaching effect will be greatly reduced. Children are usually more interested in what they have seen and things related to their lives. No matter what stage it is for learners, complicated or too simple learning content can easily reduce learners’ interest. Therefore, when choosing a course, the learner’s existing knowledge will generally be considered as much as possible, and some challenging new content will be chosen for learners. Children are in the early stage of life. For the education of children, it must pay attention to comprehensive and balanced development. Therefore, in terms of curriculum selection, it should help children’s health, language, cognition, art, science, and other aspects of comprehensive development. The principles of content selection of robotics course for children are shown in Fig 2.

As the carrier of knowledge, textbooks are the embodiment of curriculum content. To compile curriculum textbooks, many curriculum-related resources are needed, and these resources are summarized and sorted out. It brings much convenience to people’s learning. Teachers and students usually use the Internet to download and learn teaching materials. However, it is impossible to quickly and accurately find effective resources on the network due to the many teaching resources in the network and the lack of specific organization of teaching resources. The content of the textbook is mainly in the form of text. Therefore, a system of automatic document classification is constructed based on the VSM, which can automatically identify document categories and effectively improve the efficiency of resource collection and sorting.

**Fig 3:**
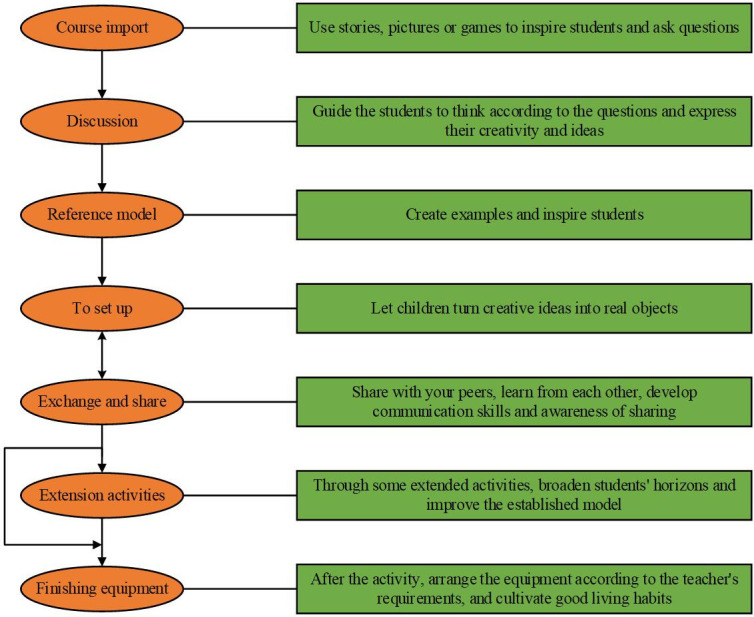
The content design of the teaching activities of robotics courses for children.

The goal of robotics courses is to cultivate children’s innovation awareness, hands-on ability, unity awareness, and thinking ability. Hence, when the curriculum is designed, it is necessary to take children as the lead and give children more hands-on practice and thinking opportunities. During the implementation of the curriculum, the main content of the teaching activities is questions raised by teachers, discussion with children, hands-on model building, exchanges, and sharing. The content design of the teaching activities of robotics courses for children is demonstrated in Fig 3.

For curriculum evaluation, it is necessary to be comprehensive, diversified, and personalized [16]. The teacher’s evaluation will affect the children’s learning mood. An objective and fair evaluation can help children to grow up better. Evaluation is not a tool for selection and elimination. For children, teachers should pay more attention to the process. Teachers should give prompt guidance to the children’s problems and praise the children’s creativity and thinking in the process. Teachers should praise children who have completed their work and encourage and guide children who have not completed their work. Detailed evaluation can promote the confidence of the children.

In robotics courses, children are the main body of the course. Children are encouraged to conduct self-evaluation and mutual evaluation, which can help them learn from each other and progress together. Parents are the most familiar with children. They can pay attention to the changes in children in time so that parents can participate in the curriculum evaluation. Parents can accompany children to learn and grow together. It is helpful for children’s learning. Each student has different talents and advantages, so when evaluating children, it is necessary to avoid using unified standards to evaluate, respect individual differences, evaluate the advantages and disadvantages of children objectively and impartially, and establish personalized evaluation standards according to the different characteristics of each child.

### 3.5 System of automatic document classification by the VSM

**Fig 4:**
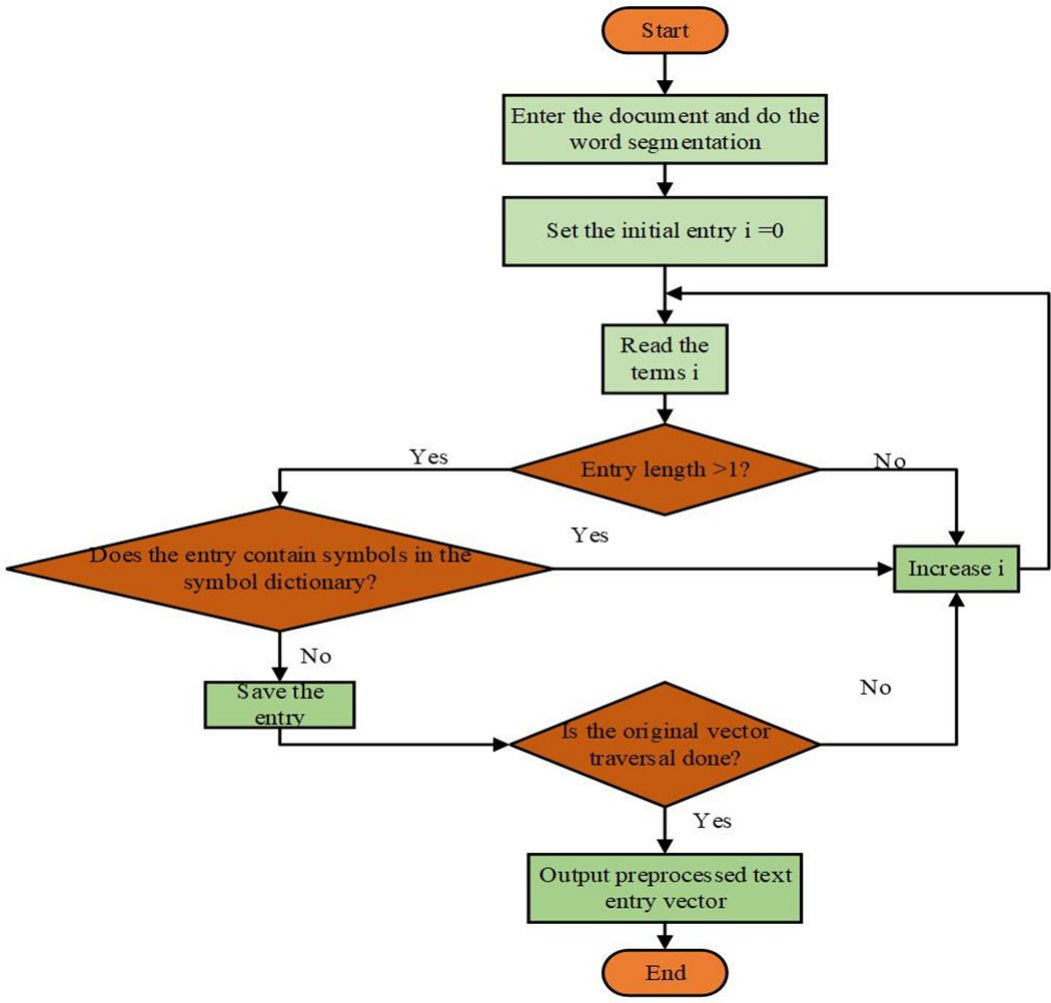
The process of building the entry vector.

VSM-based robotics course development can help better understand and generate natural language, improve interaction experience, and build more intelligent voice interaction robots. The VSM can represent natural language as a high-dimensional vector. In the development of robot courses, it is necessary to convert the questions and answers of the course into vectors and perform operations, such as similarity calculation, on these vectors to realize the robot’s understanding and answer to the questions. The text is converted into a machine-readable form by segmenting words and removing stop words from natural language text. The preprocessed text is converted into vector form. Methods, such as bag-of-words or word-vector models, can be used to convert words or sentences into vectors. Specifically, the bag-of-words model represents text as the number of times a word appears, while the word-vector model uses neural networks and other methods to produce a distributed vector representation of words. Firstly, the text needs to be preprocessed. For the VSM, the feature is usually words or phrases. Still, it is believed that the classification effect of choosing phrases as the feature is better than selecting words. So, the text is divided into words first and used as vector elements to express the text. After the Chinese word segmentation is segmented, the entry will have many separate words. These separate words reduce the accuracy and efficiency of the classification system. When preprocessing, the separate words in the text are filtered. Chinese entries containing mathematical or English symbols do not affect the accuracy of the classification system. Therefore, these entries can be ignored. The process of building the entry vector is shown in Fig 4.

In Chinese text, entries are generally used as the smallest semantic carrier [17]. There is a certain probability that the original feature space will consist of all entries in the article. The total number of Chinese entries is more than 200,000, which will form a high-dimensional feature space. For all classification algorithms, it is difficult to process high-dimensional feature spaces. The feature space’s dimension can be reduced through feature extraction, and the classification efficiency and accuracy can be improved.

The traditional VSM treats feature words as mutually independent features without considering the correlation between features. When processing text documents containing many natural languages, it will not only lose the structural information of the text but also make the document vector unable to correctly express the document’s content. Therefore, the paragraph according to the paragraph and word spacing vectors are extracted. The document’s structural characteristics can show the document’s overall thinking. Through learning the logical structure and content of the paragraph, the overall thinking of the document can be understood.

The traditional Term Frequency-Inverse Document Frequency (TF-IDF) [18] extracts document features according to the word or phrase of the document and vectorizes the document. This method cannot reflect the connection between the document word and word and phrase and phrase. A method similar to TF-IDF is used. The terms are put into separate paragraphs for weighting. Then, each paragraph in the document is analyzed separately. The equation for calculating the weight of the feature *t* in the paragraph *p*_*k*_ is shown in Eq (9).

$$val\left(t,p_{k}\right)=\frac{tf\left(t,p_{k}\right)\times \left(np_{t}/N_{p}\right)}{\sqrt{\sum_{t\in p_{k}}{\left[tf\left(t,p_{k}\right)\times \left(np_{t}/N_{p}\right)\right]}^{2}}}$$ (9)

*tf*(*t*, *p*_*k*_) represents the word frequency of the feature *t* in paragraph *p*_*k*_. *np*_*t*_ shows the number of paragraphs in the document where the feature *t* appears, and N_p is the total number of sections in the document.

The expression of the VSM of the section is shown in Eq (10).


$$p_{k}=\left(val\left(t_{1},p_{k}\right),val\left(t_{2},p_{k}\right),\cdots ,val\left(t_{n},p_{k}\right)\right)$$ (10)

The correlation between words in sentences can reflect the correlation between words in paragraphs. The closer the distance between words, the higher the correlation between words. The correlation between the two words in the section is obtained according to Eq (11).

$$R_{ij}=\frac{C_{ij}}{C_{i}+C_{j}+C_{ij}}\sum_{a=1}^{n}\;r_{a}$$ (11)

*i* and *j* represent related words in the paragraph. *C*_*i*_ shows the number of occurrences of *i* in the section. *C*_*j*_ represents the number of occurrences of *j* in the paragraph. *C*_*ij*_ indicates the number of sentences in *i* and *j* appear simultaneously in the paragraph, and *n* represents the simultaneous occurrence of the total number of *i* and *j* in sentences in the paragraph. *r*_*a*_ is the correlation coefficient, and *r*_*a*_ can be calculated according to Eq (12).

$$r_{a}=\frac{log_{2}\left[1/\left(dis+1\right)\right]}{log_{2}\left(1/S\right)}dis\leq S$$ (12)

*dis* represents the distance between *i* and *j*, and *S* is the number of words contained in the longest sentence in the paragraph.

The correlation between word and word in the sentence is applied to the paragraph vector, and the weight of the feature *t* in the paragraph *p*_*k*_ is shown in Eq (13).

$$val^{\prime }\left(t,p_{k}\right)=val\left(t,p_{k}\right)\times \sum_{j=1}^{m}\;R_{tj}$$ (13)

*m* is the number of other features that appear together with feature *t* in the paragraph. $\sum_{j=1}^{m}R_{tj}$ can be used to reflect the importance of feature *t*.

The correlation between sections is calculated by the angle cosine equation, as shown in Eq (14).


$$Sim\left(p_{i},p_{j}\right)=cos\theta =\frac{\sum_{k=1}^{n}val^{\prime }\left(t,p_{i}\right)\times val^{\prime }\left(t,p_{j}\right)}{\sqrt{\sum_{k=1}^{n}val^{\prime 2}\left(k,p_{i}\right)\times val^{\prime 2}\left(k,p_{j}\right)}}$$ (14)

The equation calculates the paragraph’s position weight is shown in Eq (15).


$$W_{pk}=\partial \times \frac{num}{\text{max}\left(num_{i}\right)}+\left(1-\partial \right)\times position_{pk}$$ (15)

0 < ∂ < 1, and the best value can be determined by experiment. *num* is the number of times *p*_*k*_ appears when the similarity of other paragraphs is greater than repeated experiments. Then, the critical value K can be obtained. The value of *position*_*pk*_ at different locations is different.

The weight of the feature *i* can be obtained according to Eq (16).

$$w_{t}^{\prime }\left(t,d\right)=\sum_{k=1}^{p}\left[val^{\prime }\left(t,p_{k}\right)\times W_{pk}\right]$$ (16)

*p* is the total number of paragraphs in the document. The weight of the document is expressed as:

$$W_{t}\left(t,d\right)=w_{t}\left(t,d\right)\times w_{t}^{\prime }\left(t,d\right)$$ (17)

*w*_*t*_ (*t*, *d*) is the weight value of the feature *i* according to the traditional TF-IDF method.

The VSM used in the automatic text classification system is shown in Eq (18).


$$\vec{d}=\left(W_{1},W_{2},\cdots ,W_{n}\right)$$ (18)

The performance of the classification system is usually evaluated by classification precision [19], recall [20], and F1 value.


$$precision=\frac{TP}{TP+FP}$$ (19)

$$recall=\frac{TP}{TP+FN}$$ (20)

$$F1=\frac{2\times precision\times recall}{precision+recall}$$ (21)

## 4. Result and discussion

### 4.1 Basic situation and result analysis of the questionnaire

**Fig 5:**
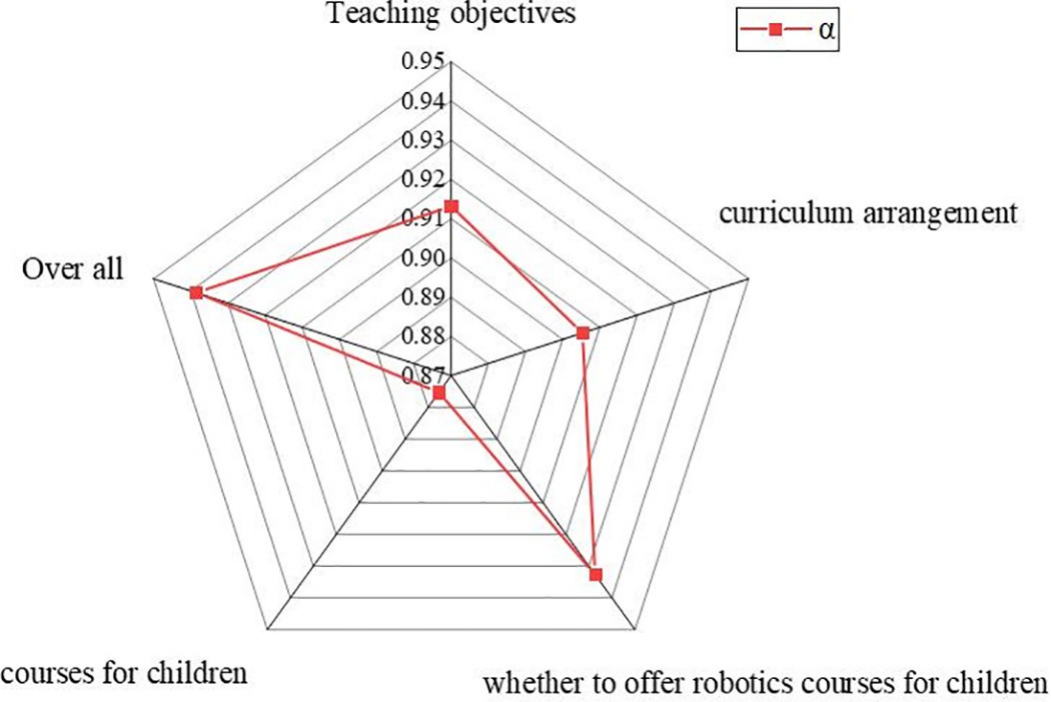
Reliability test of the questionnaire.

Cronbach’s alpha is used to test the reliability of the questionnaire, and the results are revealed in Fig 5.

Fig 5 shows that the α value of the three dimensions in teaching objectives of the kindergarten, curriculum arrangement, and whether to offer robotics courses is above 0.9, indicating that it has a good level of reliability. Regarding the attitude dimension, its α value is greater than 0.8, and its reliability is also within the allowable range. The overall reliability of the questionnaire is 0.9385, which is greater than 0.9, indicating that the overall reliability of the questionnaire is relatively high.

**Fig 6:**
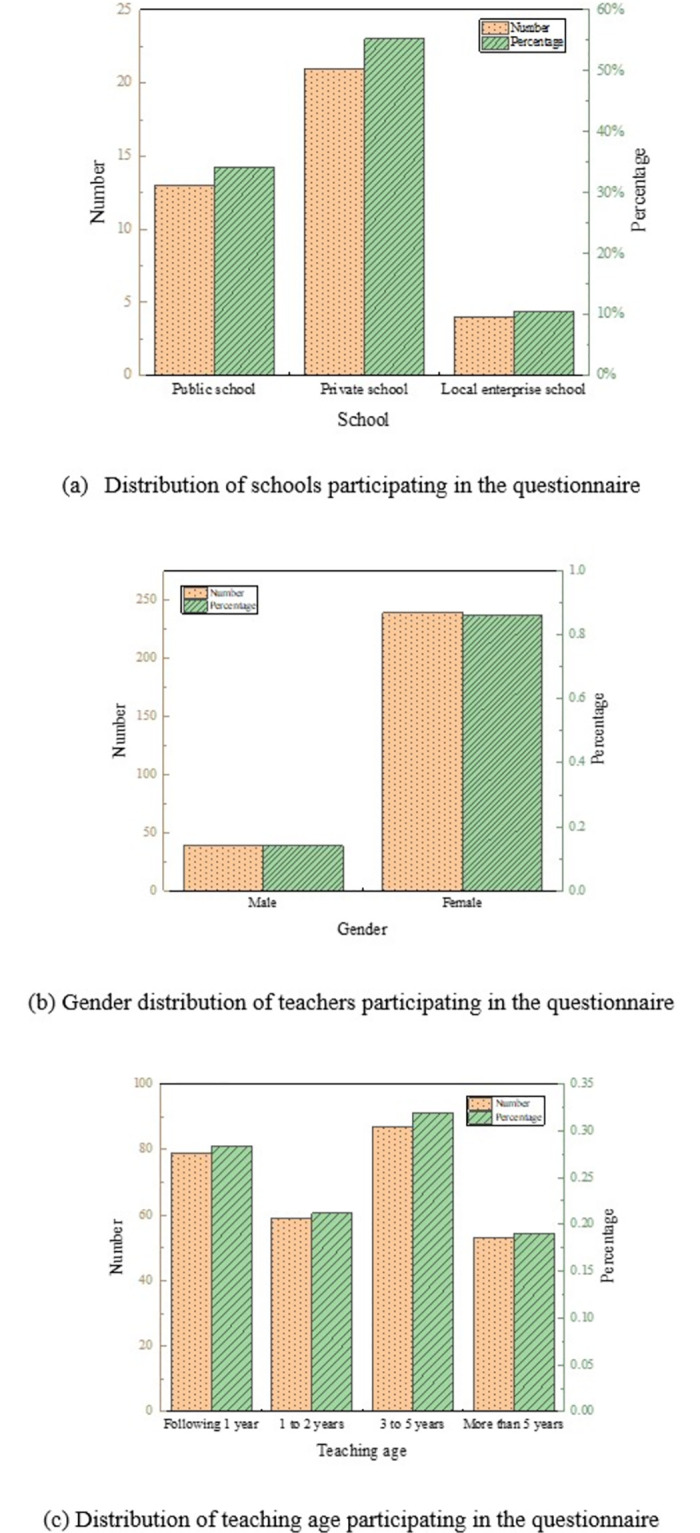
Distribution of schools and teachers participating in the questionnaire. (a) Distribution of schools participating in the questionnaire. (b) Gender distribution of teachers participating in the questionnaire. (c) Distribution of teaching age participating in the questionnaire.

The distribution of schools, the gender distribution of teachers, and the distribution of teaching age participating in the questionnaire are shown in Fig 6.

Among the schools participating in this questionnaire, private schools have the largest number of schools, with 21 schools, and local enterprise schools have the least number of schools, with only four schools. In the distribution of teachers participating in the questionnaire, the number of male teachers is only 39, which is far less than that of female teachers. This is because in kindergartens, the number of male teachers is relatively small. The number of teachers with a teaching age of 3–5 years is rather large, with 87, and the smallest number of teachers with more than five years of teaching, with 53.

**Fig 7:**
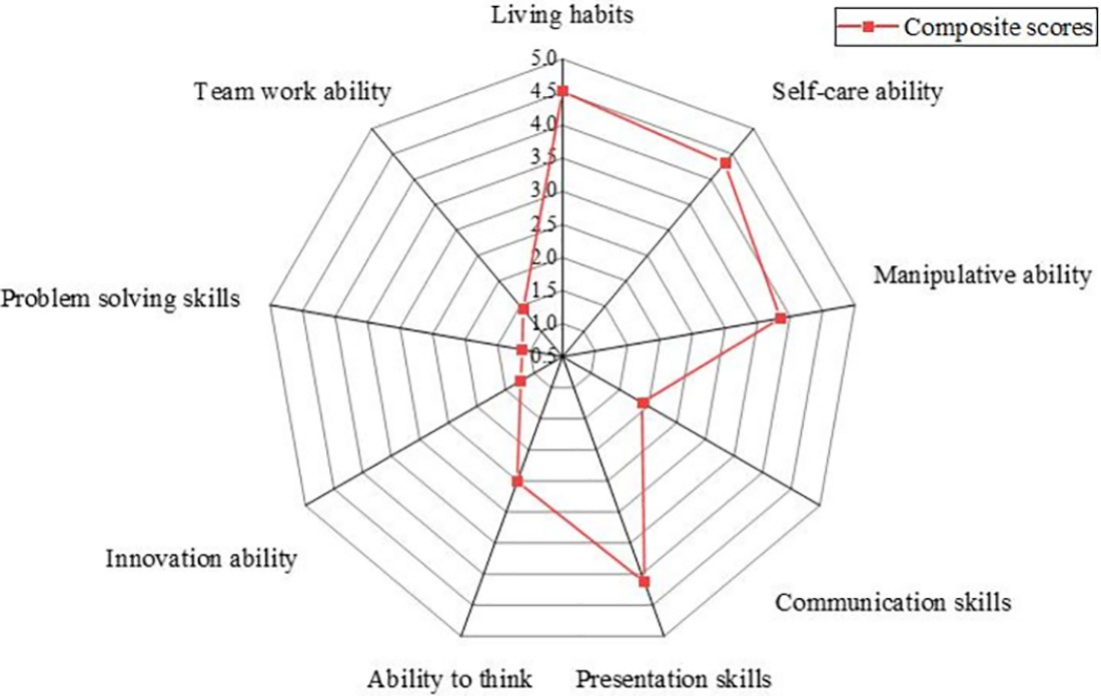
Results of the questionnaire on teaching objectives.

**Fig 8:**
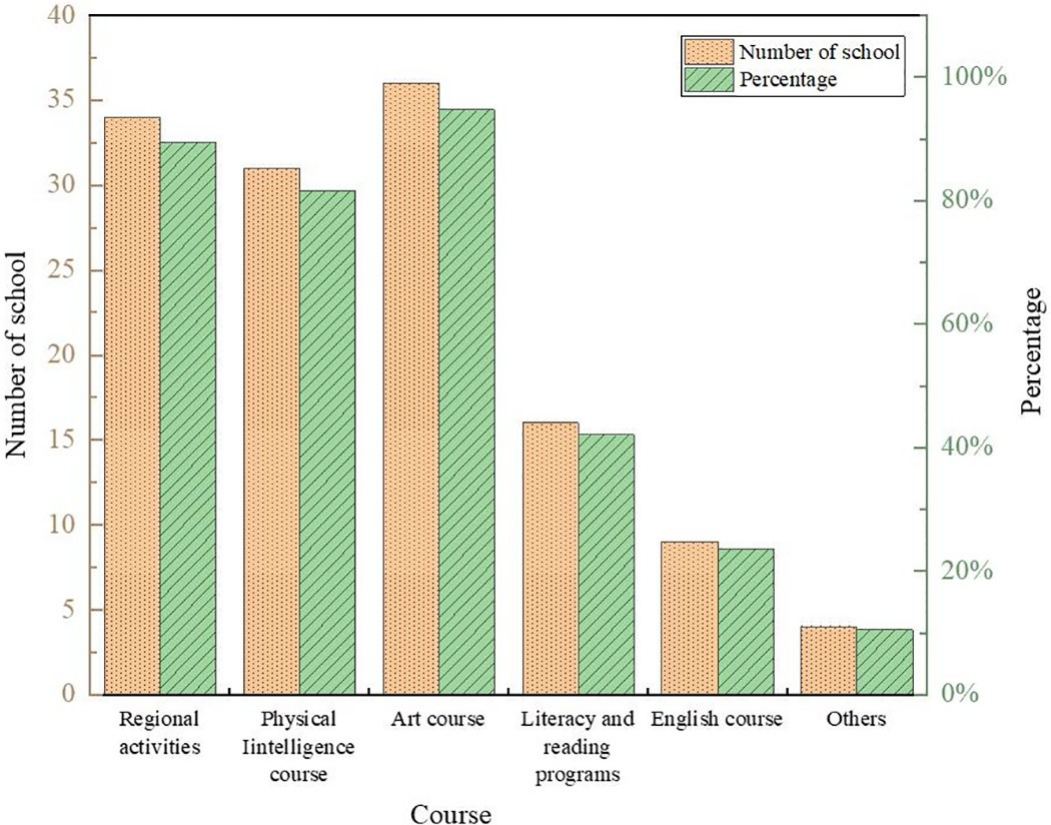
Results of courses currently offered in kindergartens.

**Fig 9:**
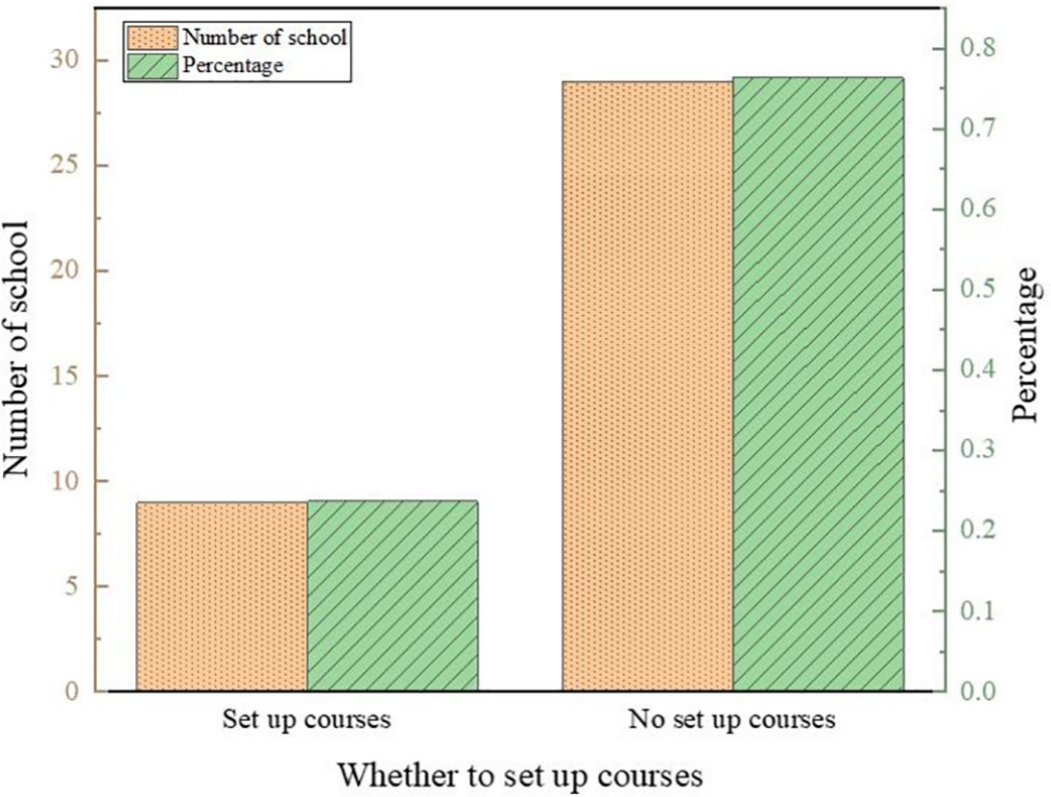
Results of whether to offer courses in kindergartens.

**Fig 10:**
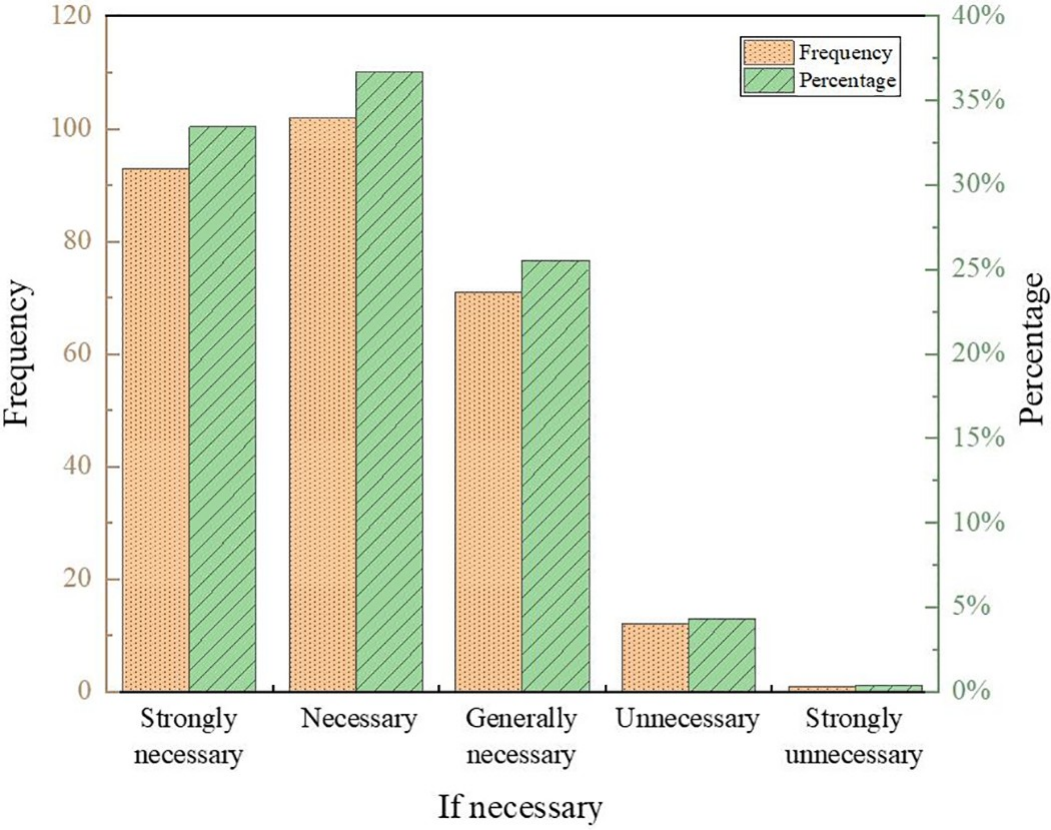
Teachers’ attitudes toward robotics courses for children.

The results of the questionnaire are shown in Figs 7–10.

The results show that kindergartens pay more attention to cultivating children’s good living habits and self-care ability, with a comprehensive score of 4.51 and 4.32, respectively, with high scores. In addition, children’s presentation skills and manipulative abilities are paid more attention to. Most kindergartens in Baoji City currently offer regional activity courses, physical intelligence courses, and art courses, accounting for 89.74%, 81.58%, and 94.74%, respectively. In contrast, literacy and reading courses, English courses, and other knowledge courses account for less than 50%, the same as the Directions of Preschool Education. Kindergartens required by China should take games as the basic activity combine education with fun and meet the needs of children’s physical and mental development. About 24% of kindergartens offer robotics courses, and 76% do not. Regarding whether a kindergarten should offer robotics courses, 33.45% of teachers think it is necessary, 36.69% of teachers believe it is necessary, accounting for 70.14%, while teachers who think it is unnecessary and strongly unnecessary only accounted for 4.68%. It indicates that most teachers support the establishment of robotics courses for children.

### 4.2 System performance evaluation

**Fig 11:**
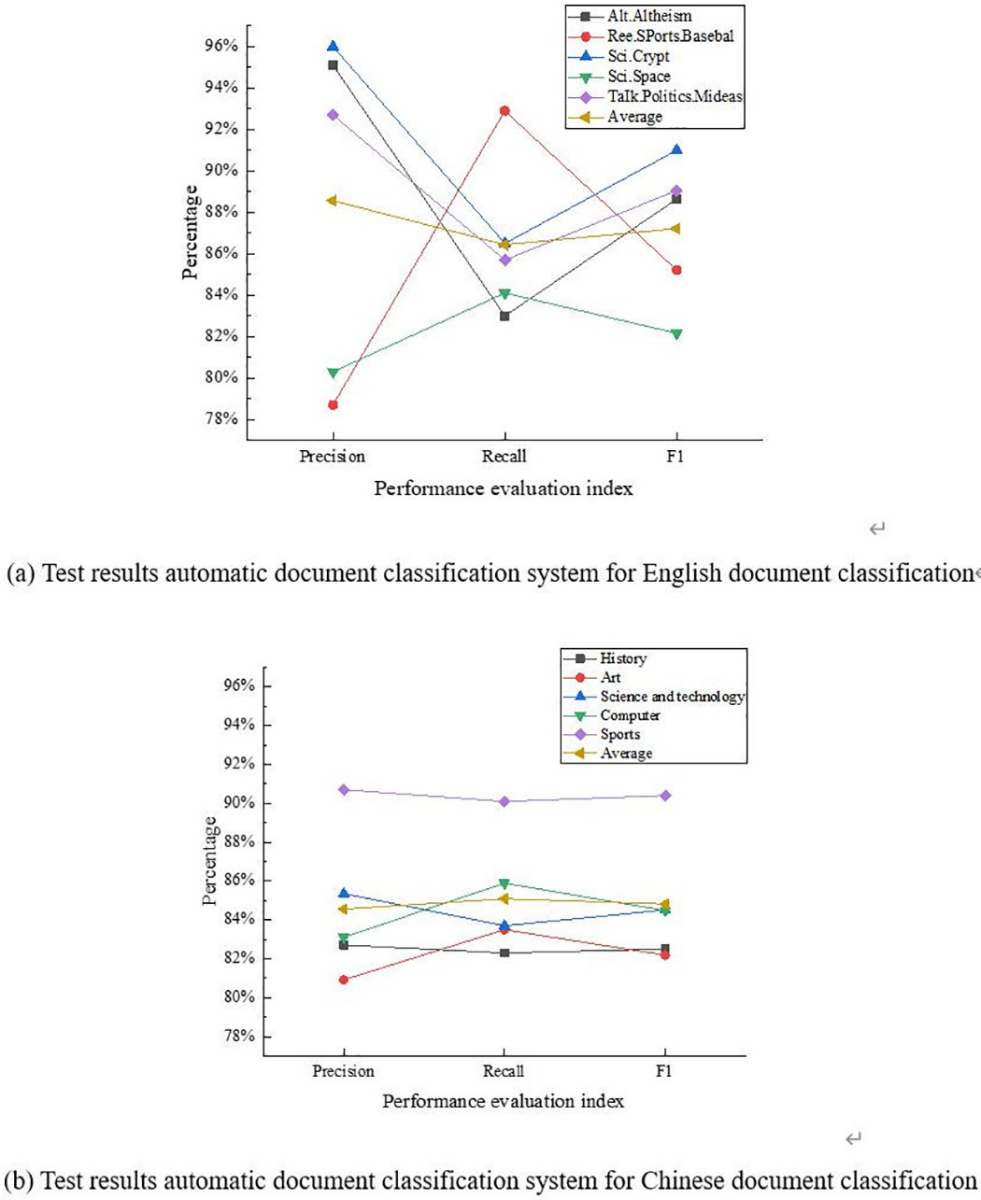
Test results of the performance of the document classification system. (a) Test results automatic document classification system for English document classification. (b) Test results automatic document classification system for Chinese document classification.

The construction of an automatic document classification system is used to classify and test 500 documents from Chinese history, art, technology, computer, and sports, and 500 English documents from Alt.Altheism, Ree.SPorts.Basebal, Sci.Crypt, Sci.Space, and TaIk.Politics.Mideas, respectively. The results of precision, recall rate, and F1 value are plotted in Fig 11.

Fig 11 indicates that the average values of the precision, recall, and F1 of the built document classification system for the classification of English documents are 88.6%, 86.0%, and 87.2%, respectively. The average values of the precision, recall, and F1 for the classification of Chinese documents are 84.5%, 85.1%, and 84.8%. The precision, recall rate, and F1 of English document classification are generally greater than those of Chinese. This is mainly due to the complexity of Chinese word segmentation.

**Fig 12:**
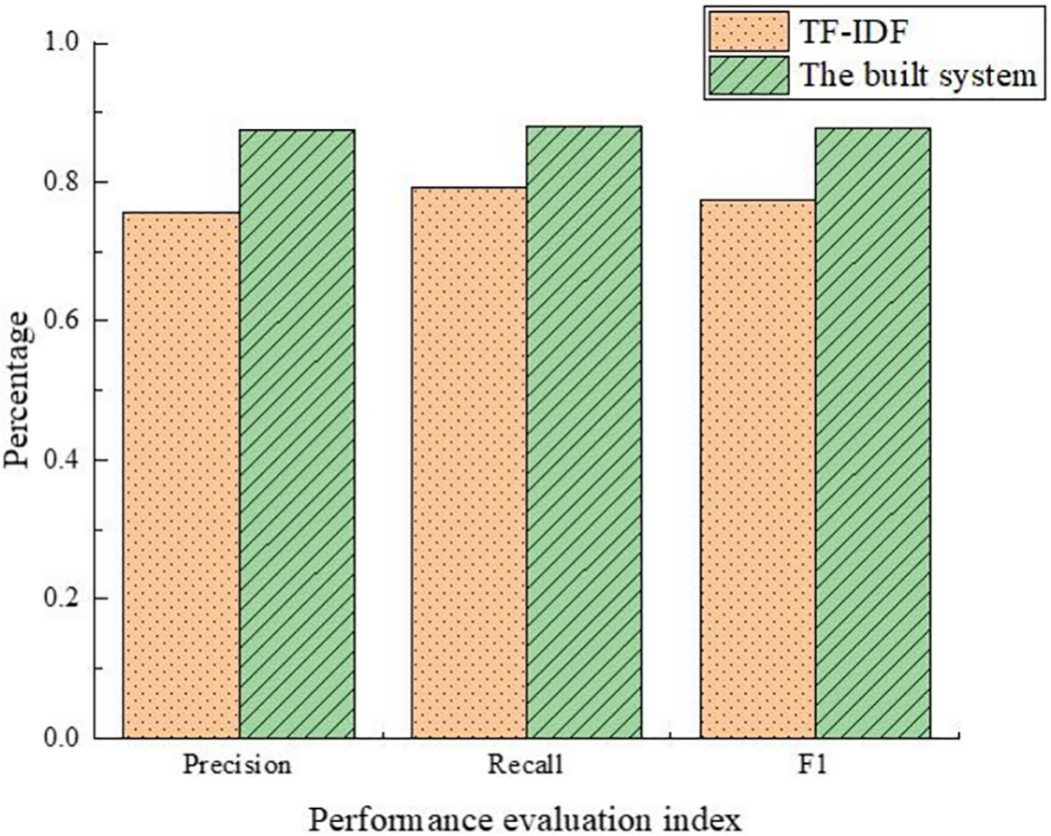
Comparison of the built automatic document classification system and the automated document classification system based on TF-IDF.

The built automatic document classification system and the traditional automatic document classification system by TF-IDF are tested on 2,000 documents. Fig 12 reveals the results.

Fig 12 shows that the classification precision, recall, and F1 value are all above 85% of the built automatic document classification system and the traditional classification system. In contrast, the classification precision, recall, and F1 value are lower than 80% of the automated document classification system based on TF-IDF. It means that the built automatic document classification system performs better than the traditional classification system based on TF-IDF.

### 4.3 Discussion

The innovation points are mainly reflected in two aspects. On the one hand, the construction of robotics courses is tried in preschool education. Most of the domestic research is based on primary and secondary schools, and the design of robot courses for primary and secondary schools is far greater than the research on preschool robot courses. Most preschool robotics courses are conducted with the help of extracurricular institutions. As a result, there is an increasing demand for robotics courses in kindergartens, while existing preschool robotics courses do not meet the actual needs. Teachers do not have suitable robotics course materials, and the existing course materials are out of touch with the real situation. Therefore, this paper designs robot courses suitable for kindergartens, hoping to provide some robot course experience and prepare kindergarten and kindergarten teachers for future robot courses. On the other hand, the concept of VSM is combined with the robot course to get rid of the previous teaching mode so that children can acquire knowledge in practice to cultivate mobility.

Kewalramani et al. (2021) [21] believed that 4–5 years old was a critical period for all aspects of young children’s development. Grasping the critical period could promote the better development of young children in the future and pave the way for the connection between kindergarten and primary school. The findings of this paper are consistent with this conclusion. Sisman et al. (2021) [22] pointed out that in terms of robot curriculum development and design, in the video of robot teaching in primary school, most of the explanation of basic knowledge was PPT screen recording, only sound. In this way, there was a lack of interest in the teaching process, and the noise of the sound would also affect the teaching effect. This is consistent with the findings of this paper. Therefore, the theme-based curriculum of robotics course education should focus on the integration of knowledge between fields. Each activity revolves around the same theme, making the activity more targeted. Carrying out relevant activities under the same theme can consolidate the indirect experience acquired by children, which is more conducive to the formation of good qualities and the improvement of children’s comprehensive ability literacy.

## 5. Conclusion

With the continuous development of intelligent robots, robotics courses have gradually been recognized by parents and students because they can improve students’ manipulative, thinking, problem-solving, teamwork, and other abilities. Firstly, a questionnaire is conducted on the courses offered by kindergartens in Baoji City. AI technology is integrated into the development process of young children’s robotics courses. Secondly, the development of robotics courses for young children is carried out through the research of four links: determination of curriculum objectives, content selection, implementation methods, and curriculum evaluation, and an automatic document classification system is constructed by the VSM to assist in the compilation of textbook content. Finally, the results of the questionnaire are studied, and the performance of the automatic document classification system is tested. The results show that kindergartens focus more on cultivating children’s good living habits and self-care abilities. About 24% of kindergartens offer robotics courses, and 76% do not. Furthermore, 70.14% of teachers support offering robotics courses for children. The built system has a better classification effect on English documents than Chinese ones. Compared with the automatic document classification system based on TF-IDF, it can be found that the built system has better performance, and its classification precious, recall rate, and F1 value are more than 85%.

Constructivism believes that in addition to considering teaching objectives and content, how to bring a constructive environment to young children is also particularly important. This paper further expands the application of constructivist theory in multimedia-assisted teaching and plays an overall guiding role in robot-assisted teaching. The research provides theoretical experience for developing robotics courses for children, which is of great practical significance. There are still some shortcomings. In conducting the survey, only one form of questionnaire is used, which is not comprehensive. Therefore, field investigation should be considered in follow-up research, and the results of the survey should be further combined to improve the performance of the built system.

## Supporting information

S1 Data (XLSX)
